# Noise induces Ca^2+^ signaling waves and *Chop*/*S-Xbp1* expression in the hearing cochlea

**DOI:** 10.1172/jci.insight.181783

**Published:** 2024-12-10

**Authors:** Yesai Park, Jiang Li, Noura Ismail Mohamad, Ian R. Matthews, Peu Santra, Elliott H. Sherr, Dylan K. Chan

**Affiliations:** 1 Department of Otolaryngology-Head and Neck Surgery,; 2 Department of Neurology, and; 3 Department of Pediatrics, Institute of Human Genetics, Weill Institute for Neurosciences, UCSF, San Francisco, California, USA.

**Keywords:** Otology, Calcium signaling, Cell stress

## Abstract

Exposure to loud noise is a common cause of acquired hearing loss. Disruption of subcellular calcium homeostasis and downstream stress pathways in the endoplasmic reticulum and mitochondria, including the unfolded protein response (UPR), have been implicated in the pathophysiology of noise-induced hearing loss. However, studies on the association between calcium homeostasis and stress pathways have been limited due to limited ability to measure calcium dynamics in mature-hearing, noise-exposed mice. We used a genetically encoded calcium indicator mouse model in which GCaMP6f is expressed specifically in hair cells or supporting cells under control of Myo15Cre or Sox2Cre, respectively. We performed live calcium imaging and UPR gene expression analysis in 8-week-old mice exposed to levels of noise that cause cochlear synaptopathy (98 db sound pressure level [SPL]) or permanent hearing loss (106 dB SPL). UPR activation occurred immediately after noise exposure, and the pattern of UPR activation was dependent on noise level, with the proapoptotic pathway upregulated only after 106 dB noise exposure. Spontaneous calcium transients in hair cells and intercellular calcium waves in supporting cells, which are present in neonatal cochleae, were quiescent in mature-hearing cochleae but reactivated upon noise exposure. Noise exposure of 106 dB was associated with more persistent and expansive intercellular Ca^2+^ signaling wave activity. These findings demonstrate a strong and dose-dependent association between noise exposure, UPR activation, and changes in calcium homeostasis in hair cells and supporting cells, suggesting that targeting these pathways may be effective to develop treatments for noise-induced hearing loss.

## Abstract 2

Loud noise exposure causes changes in calcium within cells of the inner ear and associated activation of the unfolded protein response, leading to hearing loss.

## Graphical abstract

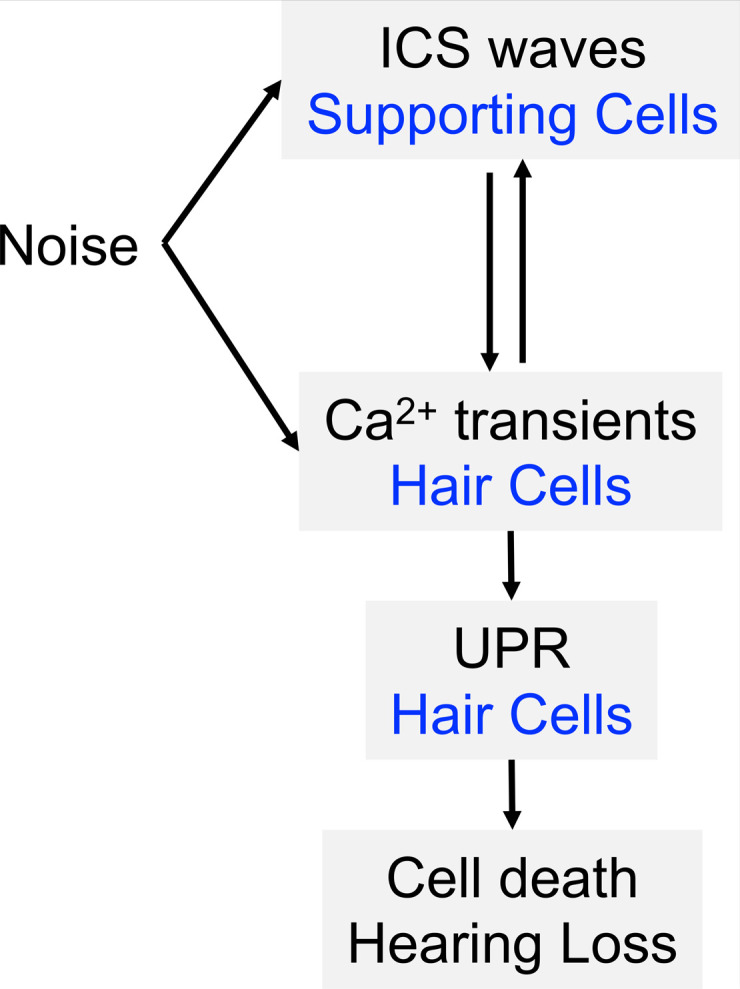

## Introduction

Noise-induced hearing loss (NIHL) affects an estimated 40 million individuals in the US, with no approved medical treatments (1). A number of cellular mechanisms have been proposed to be involved in NIHL, including oxidative stress (2, 3), JNK/ERK pathway activation (4), mitochondrial stress (5), endoplasmic reticulum (ER) stress, and the unfolded protein response (UPR) (6); targeting these pathways has identified multiple candidate drugs that prevent NIHL to varying degrees in mice: Ru360, a mitochondrial Ca^2+^ uniporter (MCU) inhibitor that reduces mitochondrial Ca^2+^ uptake and overload (7); ISRIB, an eIF2B activator that inhibits the proapoptotic PERK/CHOP pathway of the UPR (6); D-JNKI-1, a peptide inhibitor of c-Jun N-Terminal Kinase that blocks the MAPK-JNK signaling pathway (8); and N-acetyl cysteine, an antioxidant that reduces reactive oxygen species and oxidative stress (9). Of these, D-JNKI-1 and NAC have been tested in humans and have shown limited efficacy in reducing NIHL (10, 11). A better understanding of the precise mechanistic pathways by which noise induces cellular pathways in the cochlea leading to sensory hair cell death is essential to develop more effective treatments.

Many of these potential pathways — mitochondrial/oxidative stress, ER stress, and the UPR — are activated upon disruption of Ca^2+^ homeostasis. In addition to acquired hearing loss, many genetic forms of deafness involve molecules involved in Ca^2+^ flow and homeostasis in cochlear cells (12), illustrating the broad-based importance of these pathophysiologic mechanisms. Dysregulation of subcompartmental Ca^2+^ homeostasis has been directly implicated in hair cell death using an aminoglycoside model of ototoxicity in zebrafish hair cells, in which ER Ca^2+^ depletion leads to cytosolic Ca^2+^ accumulation, mitochondrial Ca^2+^ overload, and mitochondrial stress (13, 14). In mammals, both ER Ca^2+^ depletion (leading to UPR activation) and mitochondrial Ca^2+^ overload (leading to oxidative stress) have been implicated in hearing loss (6, 7). We have shown that disruption of TMTC4 — an ER-resident, hair cell–specific gene implicated in progressive hearing loss in mice and humans (15) — causes ER Ca^2+^ depletion and UPR activation, and we have shown that noise exposure causes UPR activation (6). Importantly, we found that targeting the UPR with ISRIB, a small molecule activator of eIF2B, reduces NIHL and cochlear synaptopathy (6, 16). On the other hand, genetic or pharmacologic disruption of MCU, which reduces mitochondrial Ca^2+^ accumulation, also protects against NIHL (7). Despite this strong evidence that disruption of Ca^2+^ homeostasis in hair cells can lead to hair cell death through ER and/or mitochondrial stress pathways, it is not clear how, and whether, noise trauma directly causes this Ca^2+^ dysregulation in hair cells.

One possibility is that mechanical trauma or noise exposure affects Ca^2+^ homeostasis through induction of intercellular Ca^2+^ signaling (ICS) waves in supporting cells. ICS waves have been studied extensively in the central nervous system; they propagate across glial networks, where they respond to mechanical and excitotoxic trauma and mediate neuronal repair, death, and migration (17, 18). Noise and mechanical trauma have also been suggested to induce ICS waves across supporting cell networks in the cochlea (19), but not hair cells; ICS waves are dependent on Cx26 to propagate, and Cx26 is only expressed in supporting cells (20). These changes in Ca^2+^ flux have been implicated in downstream cellular signaling cascades, including activation of the ERK pathway (21) as well as the UPR (6). In the neonatal cochlea, ICS waves occur spontaneously in supporting cells of the inner sulcus (IS) and outer sulcus (OS) in the late stages of cochlear development, synchronizing inner hair cell (IHC) firing (22, 23). ICS waves can also be triggered in neonatal cochleae by external ATP or direct mechanical trauma (19, 21, 24). Though spontaneous ICS activity was initially thought to become quiescent after the onset of hearing (23), subsequent studies have shown limited evidence of spontaneous (25) and noise-evoked (26) ICS waves in the adult mouse and gerbil cochlea, respectively. The role of these supporting cell ICS waves in the inner ear’s response to noise, however, is poorly understood.

Despite these studies demonstrating links between noise exposure and ICS signaling in supporting cells in mechanically traumatized neonatal cochlea (19), between Ca^2+^ transients in hair cells and cell death in zebrafish aminoglycoside ototoxicity (13, 14), and between ER and mitochondrial stress and noise exposure in mice (6, 7), comprehensive evaluation of the Ca^2+^ and stress pathways by which noise exposure leads to hearing loss has been limited due to the absence of a single experimental model in which dynamic cellular processes can be observed live in the mature, hearing cochlea after physiologically relevant noise exposure. Such work would require a single model system in which the effect of noise exposures on Ca^2+^ homeostasis in hair cells and supporting cells can be directly correlated with the effect of the same noise exposures on downstream preapoptotic pathways, such as the UPR, and hearing loss.

In this study, we sought to evaluate the hypothesis that excessive noise induces Ca^2+^ homeostatic changes in both hair cells and supporting cells, leading ultimately to proapoptotic UPR activation. In order to do this, we have developed a live-imaging model of the mature, hearing cochlea, enabling direct visualization of cytosolic Ca^2+^ dynamics in hair cells and supporting cells after physiologically relevant noise exposure. This model allows us to measure 3 elements of the Ca^2+^ dysregulation/UPR axis in response to the same set of physiologically relevant noise exposures that cause either temporary or permanent shifts in hearing thresholds: (a) UPR gene expression; (b) Ca^2+^ transients in hair cells; and (c) ICS waves in supporting cells. Evidence of these 3 phenomena provides support that they are all connected in the early response of the cochlea to acoustic overstimulation.

## Results

### UPR expression after NIHL.

**Figure 1:**
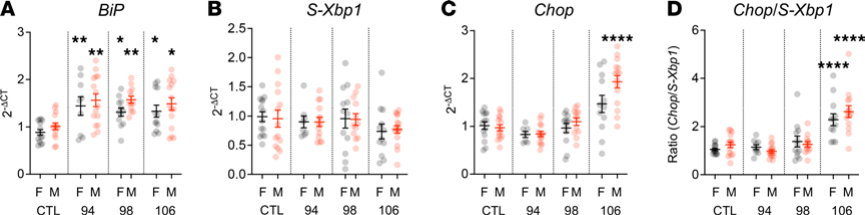
Noise exposure induces UPR upregulation in the cochlea in vivo. To investigate the sound level dose dependence of UPR activation after acoustic overstimulation, we exposed 8-week-old female (F) and male (M) WT CBA/J mice to 8–16 kHz octave-band noise for 2 hours at 94, 98, and 106 dB, levels that respectively induce no hearing loss, temporary threshold shift (TTS) with cochlear synaptopathy, and permanent threshold shift (PTS) with hair cell death. (**A**–**D**) The 2 cochleae from each animal were harvested and pooled for qPCR measurement of *BiP* (**A**), *S-Xbp1* (**B**), *Chop* (**C**), and ratio of *Chop/S-Xbp1* (**D**) mRNA expression using the 2^–ΔCt^ method relative to *Gapdh* expression and normalized to control (unexposed) levels. Data were cleaned by removing outliers (ROUT method, *q* = 1%; 6 of 412 (1.5%) data points removed) and compared with 1-way ANOVA with Dunnett’s test for multiple comparisons against control for each condition. Data are presented as means ± SEM, with individual animals shown as dots. *P < 0.05; **P < 0.01; ****P < 0.0001. *n* = 14 mice (CTL, F); 14 (CTL-M); 9 (94 dB, F); 15 (94 dB, M); 12 (98 dB, F); 12 (98 dB, M); 12 (106 dB, F); 15 (106 dB, M).

We first investigated the response of the UPR to varying levels of noise exposure. Eight-week-old male and female WT CBA/J mice were exposed to 8–16 kHz octave-band noise for 2 hours at 3 levels: 94 dB sound pressure level (SPL), which does not cause any threshold shift; 98 dB SPL, which causes temporary threshold shift (TTS) and cochlear synaptopathy (16); and 106 dB SPL, which causes permanent threshold shift (PTS) and hair cell death (6) (Supplemental Figures 1 and 2; supplemental material available online with this article; https://doi.org/10.1172/jci.insight.181783DS1). Cochleae were extracted from mice 2 hours after completion of noise exposure, and expression levels of 3 UPR marker genes — *BiP*, *Chop*, and *S-Xbp1* — were measured by quantitative PCR (qPCR) (Figure 1). *BiP*, a marker for general activation of the UPR, was significantly elevated with all noise exposure levels, demonstrating that noise exposure upregulated the UPR. No significant changes in *S-Xbp1*, a specific marker for the prohomeostatic arm of the UPR, were seen compared with control. *Chop*, a marker of the proapoptotic arm of the UPR, was elevated only in male mice exposed to 106 dB SPL. However, when the ratio of *Chop*/*S-Xbp1*, which indicates a shift in the balance of the UPR toward apoptosis (27, 28), was compared across conditions, significant elevation of this ratio was seen in both male and female mice exposed to 106 dB SPL noise. These results demonstrate that, although the UPR is activated overall with any noise exposure (as indicated by *BiP*), noise levels that cause PTS and hair cell loss are associated specifically with significant elevation of the *Chop*/*S-Xbp1* ratio. Overall, no statistically significant differences were seen relating to biological sex; for this reason, all subsequent experiments were pooled across sexes.

In addition to qPCR for UPR marker genes, we measured the expression of 84 UPR genes using an mRNA expression panel after 106 dB SPL noise exposure compared with unexposed controls. This showed that *Ddit3* (*Chop*) (1.92-fold increase, *P* = 0.006) as well as *Hspa5* (*BiP*; 2.21-fold increase, *P* = 0.01) were statistically significantly upregulated in mice exposed to 106 dB SPL noise, consistent with single-gene qPCR findings (Figure 1). Among other genes on the panel, *Cepbp* (5.51-fold increase, *P* < 0.0001), which was previously implicated as an overexpressed gene in the proteotoxic stress response during NIHL (29), was most markedly upregulated. The findings from this panel were from bulk cochleae but are consistent with analysis of hair cell–specific data from prior RNA-Seq experiments obtained from mice exposed to 105 dB SPL noise, very similar to the 106 dB SPL noise-exposure protocol used in the current study (30) (umgear.org). These data show that, in purified outer hair cells (OHCs), both *Ddit3* (1.58-fold overexpression, *P* = 0.0077) and *Cebpb* (3.24-fold increase, *P* = 0.00016) were significantly upregulated by noise (Supplemental Figure 3A). In contrast, purified supporting cells from the same dataset show statistically significant noise-induced upregulation of *Cebpb* (2.98-fold increase, *P* = 0.005) but not *Ddit3* (1.32-fold increase, *P* = 0.15; Supplemental Figure 3A). Finally, upregulation of *Ddit3*/*Chop* in specific cell types within the cochlea was examined by whole-mount immunohistochemistry, which showed that 106 dB SPL noise caused upregulation of *Chop* in OHCs, IHCs, and inner pillar cells (Supplemental Figure 3B).

**Figure 2:**
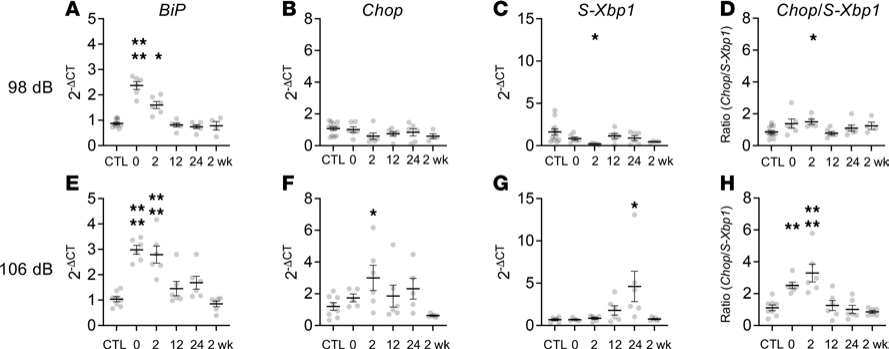
Noise exposure induces rapid UPR upregulation in the cochlea in vivo. (**A**–**H**) To investigate the temporal evolution of the UPR after acoustic overstimulation, we exposed 8-week-old male WT CBA/J mice to 8–16 kHz octave-band noise for 2 hours at 98 dB SPL (**A**–**D**), which induces TTS, or 106 dB SPL (**E**–**H**), which induces PTS. The 2 cochleae from each animal were harvested and pooled at the indicated time points after noise exposure for qPCR measurement of *BiP*, *S-Xbp1*, and *Chop* mRNA expression using the 2^–ΔCt^ method relative to *Gapdh* expression and normalized against control (unexposed) levels. Data were cleaned by removing outliers (ROUT method, *q* = 1%; 9 of 312 (2.9%) data points removed) and compared with 1-way ANOVA with Dunnett’s test for multiple comparisons against control for each condition. Data are presented as means ± SEM, with individual animals shown as dots. *P < 0.05; **P < 0.01. *n* = 12 mice (CTL, 98 dB); 8 (CTL, 106 dB); 6 (0, 2, 12, 24 hours, 98 dB and 106 dB); 6 (2 weeks, 106 dB); 4 (2 weeks, 98 dB).

We next investigated the time course of UPR activation after 98 and 106 dB SPL noise exposure, our 2 primary models for TTS and PTS, respectively. Cochleae were harvested at 0, 2, 12, and 24 hours after completion of the 2-hour noise exposure, as well as 2 weeks later, and compared with control unexposed animals (Figure 2). Elevation in *BiP* was seen immediately after noise exposure, followed by changes in *Chop* and *S-Xbp1*. Elevation in *Chop*/*S-Xbp1* ratio peaked at 2 hours after noise exposure and was more pronounced in mice exposed to the louder 106 dB SPL noise. These findings demonstrate that UPR gene expression changes are an early and dose-dependent response to noise exposure in mice.

### Live Ca^2+^ imaging in hair cells and supporting cells in the neonatal cochlea.

We then sought to use these same noise-exposure models — the 98 dB and 106 dB (8–16 kHz octave-band noise) models, which are associated with TTS (and UPR activation without shift toward apoptosis) and PTS (with proapoptotic UPR activation), respectively — and directly visualize Ca^2+^ dynamics in the organ of Corti. However, live Ca^2+^ imaging had previously been performed primarily in neonatal cochlear cultures, which cannot be stimulated with sound, and prior instances of Ca^2+^ imaging in the adult cochlea (25, 26) used exogenous dyes that did not sufficiently label hair cells. We therefore developed an acute explant preparation of the temporal bone from juvenile, mature-hearing mice (31) expressing the cytosolic Ca^2+^ indicator GCaMP6f in a Cre-dependent manner in supporting cells (driven by Sox2Cre) or hair cells (driven by Myo15Cre). This preparation was similar to one previously described that demonstrated ICS waves in the adult mouse using exogenous Fluo-4-AM dye for Ca^2+^ sensing (25) but, instead, uses a genetically encoded Ca^2+^ indicator to further expedite imaging after euthanasia and enable both hair-cell-specific and supporting-cell-specific labeling.

**Figure 3:**
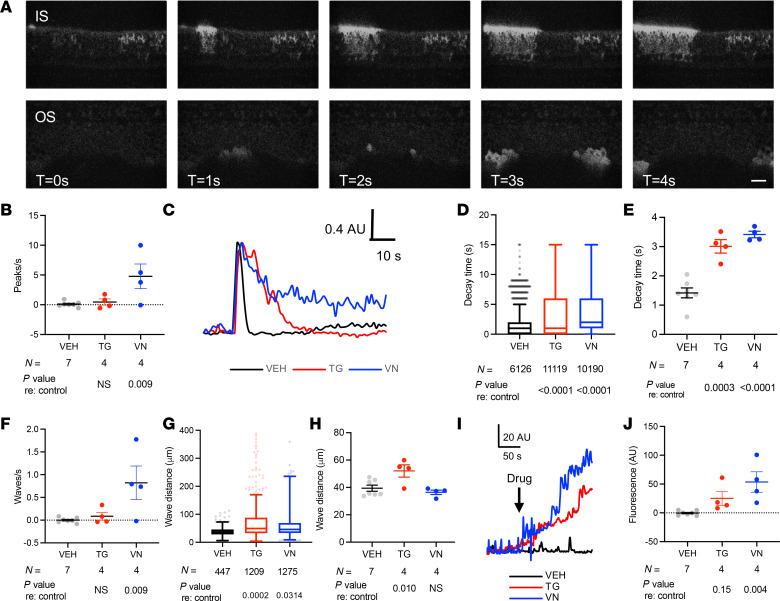
Ca^2+^ activity in neonatal cochlear supporting cells. (**A**) Live imaging of Sox2Cre-GCaMP6f neonatal cochlea. In supporting cells of the inner sulcus (IS) and outer sulcus (OS) of the neonatal cochlea, spontaneous intercellular Ca^2+^ waves are observed. Time interval between successive images is 1 second. Scale bar: 20 μm. Representative video also shown in Supplemental Video 1. (**B**) Change in number of Ca^2+^ peaks per second after drug treatment. Compared with baseline (media only), no change in frequency of Ca^2+^ peaks is seen with vehicle (VEH, black) or 1 μM thapsigargin (TG, red); 5 μM vanadate (VN, blue) induced increased Ca^2+^ peak activity. (**C**–**E**) Ca^2+^ decay time with drug treatment. Fluorescence levels (**C**) and peak decay time at the individual peak (**D**), or cochlea (**E**) level demonstrate significant prolongation of return to baseline Ca^2+^ levels in the presence of TG or VN. In **C**, peak height is normalized to maximum amplitude for each peak. (**F**) Change in number of ICS waves per second after drug treatment. No change in frequency of ICS waves is seen with VEH or TG; VN induced more ICS activity. (**F** and **G**) ICS wave propagation distance with drug treatment. ICS waves traveled significantly farther in the presence of TG, but not VN, when compared at the individual wave (**G**) or cochlea (**H**) level. (**I** and **J**) Change in steady-state Ca^2+^ after drug treatment. (**I** and **J**) Fluorescence levels and mean amplitude over a 300-second recording period, representing steady-state Ca^2+^ level across the entire cochlea, increased after TG and VN, but not VEH application. (**B**, **E**, **F**, **H**, and **J**) Data are shown as mean ± SEM, with individual cochlea-level values as dots. Groups were compared with 1-way ANOVA with Dunnett’s test for multiple comparisons against control for each condition, with *P* values as indicated. Sample sizes tested enabled detection of effect size > 1.98× SD with 80% power. (**D** and **G**) Tukey plots (box: first quartile/median/third quartile; whiskers: 10th and 90th percentile; dots: individual points outside the whiskers) representing all peaks (**D**) or waves (**G**) measured under the indicated conditions. *P* values are as indicated for pairwise comparisons versus control (VEH) on 2-tailed unpaired Student’s *t* test. AU, arbitrary units.

To validate this model, we first confirmed expression of GCaMP6f and validated its ability to detect Ca^2+^ activity in supporting cells and hair cells in neonatal mice. Neonatal cochlear cultures from Sox2Cre-GCaMP6f mice expressed GCaMP6f in supporting cells, but not hair cells (Figure 3), and exhibited spontaneous ICS waves identical to those seen using exogenous fluorophores: FURA-2 (19) and Fluo-4 (22, 25). ICS waves were observed in Kölliker’s organ at the IS as well as in OS (Supplemental Video 1 and Figure 3A). Overall, ICS waves propagated at 15.5 ± 0.5 μm/s (mean ± SEM from 128 waves in 15 cochleae) in the IS and 27.9 ± 0.4 μm/s (*n* = 914 waves) in the OS and occurred at a rate of 0.03 waves/s (IS) and 0.20 waves/s (OS). Drugs that prevent cytosolic Ca^2+^ clearance — vanadate, which blocks extrusion through plasma membrane Ca^2+^-ATPase (PMCA), and thapsigargin, which blocks ER reuptake through sarco/endoplasmic reticulum Ca^2+^-ATPase (SERCA) — affected both single-cell–level Ca^2+^ peaks and ICS characteristics. Vanadate (5 μM), but not 1 μM thapsigargin, increased the frequency of Ca^2+^ peaks and ICS waves (Figure 3, B and F). Both drugs significantly increased the decay time for the cytosolic Ca^2+^ peak to return back to baseline (Figure 3, C–E); thapsigargin, but not vanadate, increase the distance of ICS wave propagation (Figure 3, G and H). Finally, vanadate, but not thapsigargin, increased steady-state cytosolic Ca^2+^ levels (Figure 3, I and J). In contrast to these effects of vanadate and thapsigargin, tunicamycin, which induces ER stress but does not directly affect ER Ca^2+^ dynamics, had no effect on Ca^2+^ activity in neonatal cochleae other than a small increase in decay time (Supplemental Figure 4). Taken together, these findings suggest that supporting-cell–specific GCaMP6f signal in the Sox2Cre-GCaMP6f model is accurately representing ICS wave activity.

**Figure 4:**
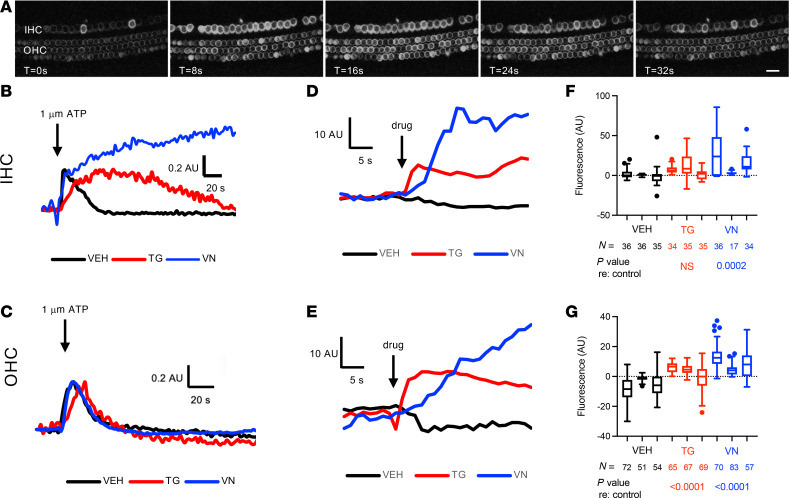
Ca^2+^ activity in neonatal cochlear hair cells. (**A**) Live imaging of Myo15Cre-GCaMP6f neonatal cochlea. No spontaneous Ca^2+^ activity was seen in hair cells of the neonatal cochlea. However, application of 1 μm ATP (applied after the leftmost panel) induced an increase in cytosolic Ca^2+^ with subsequent return to baseline in both inner hair cells (IHCs) and outer hair cells (OHCs). Time interval between successive images was 8 seconds. Scale bar: 20 μm. Representative video also shown in Supplemental Video 2. (**B** and **C**) ATP-induced Ca^2+^ peaks. In an individual cochlear IHC (**B**), bath application of 1 μm ATP (arrow) induced an increase in Ca^2+^ in the presence of vehicle (VEH, black), thapsigargin (TG, red), and vanadate (VN, blue), with prolonged onset and decay time with TG and prolonged elevation with no return to baseline in the presence of VN. An OHC (**C**) was also responsive to ATP; however, TG and VN had minimal effects on return to baseline. For both **B** and **C**, peak height was normalized to maximum amplitude of the initial ATP-induced peak. (**D**–**G**) Change in Ca^2+^ after drug treatment. Treatment with TG (red) and VN (blue) induced initial increases in Ca^2+^, as seen in mean fluorescence tracings across IHC (**D**) and OHC regions (**E**) immediately after drug treatment (arrow). Comparison of steady-state Ca^2+^ levels after drug treatment (**F** and **G**) showed that TG induced a significant persistent increase in Ca^2+^ in OHCs (**G**) but not IHCs (**F**), where VN increased steady-state Ca^2+^ in both IHCs and OHCs. (**F** and **G**) Tukey plots (box: first quartile/median/third quartile; whiskers: 10th and 90th percentile; dots: individual points outside the whiskers) representing all cells measured in individual cochleae under the indicated conditions. Groups were compared with 2-way ANOVA to detect treatment or cochlea-specific differences in fluorescence. *P* values indicate treatment effect for the indicated drug compared with control. Sample sizes tested enabled detection of effect size > 3.07× SD with 80% power. AU, arbitrary units.

We then examined neonatal cultures from Myo15Cre-GCaMP6f mice, which express GCaMP6f in hair cells with scant off-target labeling (Figure 4A and Supplemental Video 2). Minimal spontaneous activity was observed (Supplemental Video 2); occasional spontaneous Ca^2+^ transients were observed in IHCs, but these never propagated as ICS waves, consistent with the fact that hair cells do not express Connexin 26 necessary for the direct and paracrine signaling that underlies wave propagation (20). Application of ATP, however, induced a large cytosolic Ca^2+^ transient in both IHCs and OHCs, demonstrating intact purinergic responses; clearance of these ATP-induced Ca^2+^ peaks was sensitive to vanadate and thapsigargin in IHCs but not OHCs (Figure 4, B and C). Application of thapsigargin and vanadate induced initial increases in Ca^2+^ in both IHCs and OHCs (Figure 4, D and E); however, steady-state Ca^2+^ was only significantly raised by thapsigargin in OHCs, whereas vanadate increased steady-state Ca^2+^ in both types of hair cells (Figure 4, F and G), illustrating distinct patterns of Ca^2+^ homeostasis in IHCs and OHCs.

### Live Ca^2+^ imaging in hair cells and supporting cells in the mature, hearing cochlea.

**Figure 5:**
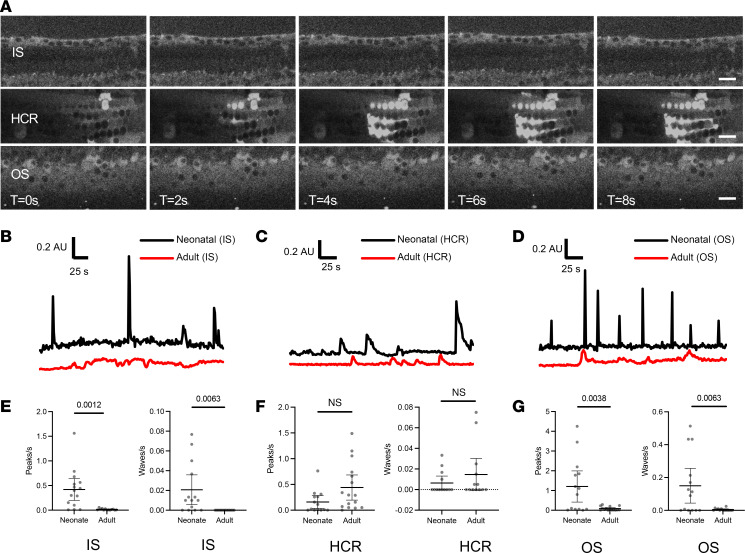
Ca^2+^ activity in adult cochlear supporting cells. (**A**) Live imaging of Sox2Cre-GCaMP6f adult cochlea. Spontaneous Ca^2+^ activity was evaluated in supporting cells of the adult cochlea within the inner sulcus (IS), hair cell region (HCR), and outer sulcus (OS). Time interval between successive images is 2 seconds. Scale bar: 20 μm. Representative video shown in Supplemental Video 4. (**B**–**G**) Activity in neonatal versus adult cochlea. Compared with neonatal cochlea (black), single cells in the IS (**B**) and OS (**D**) of the adult cochlea (red) showed reduced spontaneous Ca^2+^ peak activity. A supporting cell in the adult HCR (**C**) showed comparable spontaneous activity to neonatal cochlea. Number of peaks and waves per second was significantly higher in neonatal versus adult cochlea in both IS (**E**) and OS (**G**) regions and was not significantly different in the HCR (**F**). (**E**–**G**) Data are shown as mean ± SEM with individual values in gray. *P* values as indicated (black bars) on pairwise comparison between neonatal and adult cochlea using 2-tailed, unpaired Student’s *t* test. *n* = 15 (neonate) and 16 (adult) cochleae. AU, arbitrary units.

Having established the ability to perform hair cell– and supporting cell–specific live cytosolic Ca^2+^ imaging in neonatal cochlea, we moved to our juvenile (7- to 8-week-old), mature-hearing cochlear preparation, which enables imaging of the 8–10 kHz region of the cochlea (31, 32). GCaMP6f-expressing mice have similar baseline hearing thresholds and response to 98 dB and 106 dB SPL noise exposures as WT CBA/J mice (Supplemental Figure 5). GCaMP6f expression was visible in both hair cells (in Myo15Cre-GCaMP6f mice) and supporting cells (in Sox2Cre-GCaMP6f mice), and cells remained stable in shape and size, with no steady-state changes in cytosolic Ca^2+^ levels over the recording period, suggestive of overall cellular health in this explant preparation (Supplemental Videos 3 and 4). There were no spontaneous intracellular Ca^2+^ transients seen in hair cells (Supplemental Video 3). In supporting cells, which exhibited robust spontaneous activity in both IS and OS regions of the neonatal cochlea, reduced spontaneous activity was observed in these regions of the mature-hearing cochlea, though some activity was seen in Deiters’ cells (Supplemental Video 4 and Figure 5). This is consistent with prior report, which demonstrated quiescence of spontaneous ICS wave activity after the onset of hearing around P14 (23). Overall, these findings demonstrate that the mature-hearing cochlear explant preparation from Myo15Cre-GCaMP6f and Sox2Cre-GCaMP6f is healthy and enables detection of cytosolic Ca^2+^ but that overall Ca^2+^ activity is quiescent under control unexposed conditions.

### Noise exposure activates ICS activity in cochlear supporting cells.

**Figure 6:**
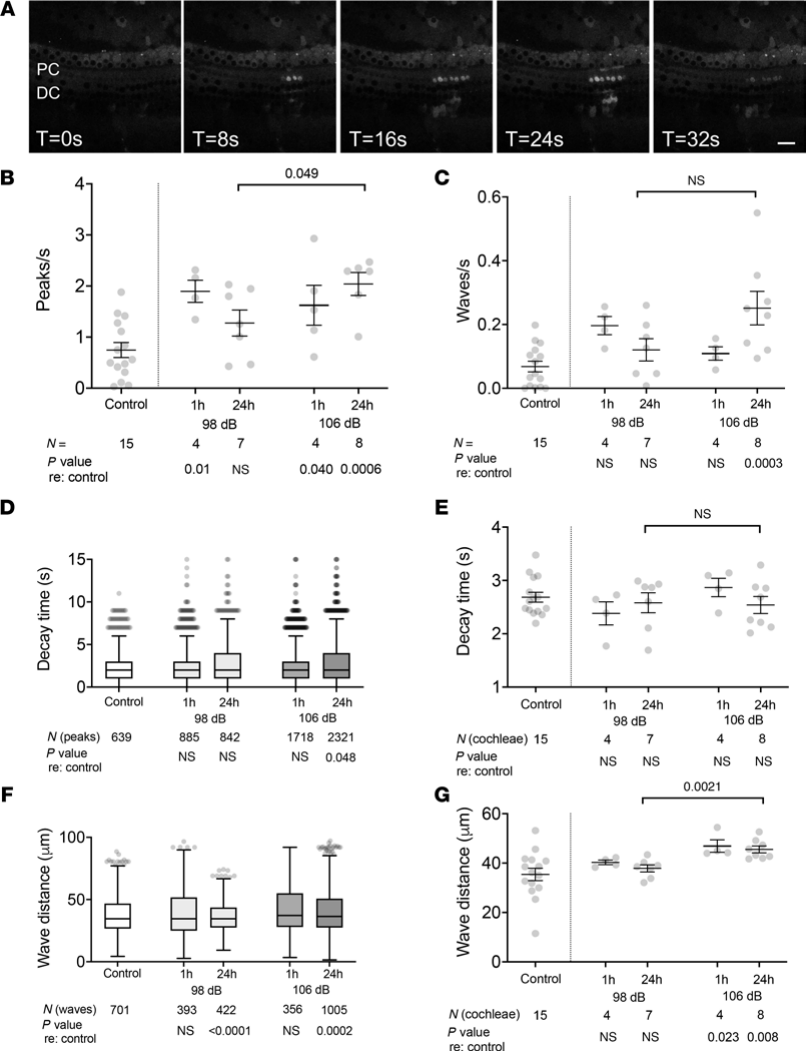
Ca^2+^ activity in adult cochlear supporting cells. (**A**) Live imaging of noise-exposed Sox2Cre-GCaMP6f adult cochlea. Preexposure to 98 dB SPL noise induces ICS wave activity in supporting cells of the adult cochlea, including pillar cells (PC) and Dieters’ cells (DC). Time interval between successive images is 8 seconds. Scale bar: 20 μm. Representative video also shown in Supplemental Video 5. (**B** and **C**) Effect of noise exposure on Ca^2+^ peak and ICS wave activity. Compared with control unexposed cochleae, cochleae from mice exposed to 98 dB as well as 106 dB noise showed increased number of Ca^2+^ peaks (**B**) and ICS waves (**C**) 1 hour after beginning of noise exposure. Twenty-four hours after completion of noise exposure, 98 dB–exposed mice had no significant increase in either Ca^2+^ peaks or ICS waves compared with control, whereas 106 dB–exposed mice had persistent elevation in both Ca^2+^ peak activity and ICS waves. (**D** and **E**) Effect of noise exposure on Ca^2+^ peak decay time. Ca^2+^ peak decay time is compared under the indicated conditions at the individual peak (**D**) or cochlea (**E**) level. (**F** and **G**) Effect of noise exposure on ICS wave propagation distance. Distance traveled for ICS waves, compared at the individual wave (**F**) and cochlea (**G**) level was increased after 106 dB but not 98 dB noise exposure. (**B**, **C**, **E**, and **G**) Data are shown as mean ± SEM, with individual values in gray. Sample size refers to the number of individual cochleae. (**D** and **F**) Tukey plots (box: first quartile/median/third quartile; whiskers: 10th and 90th percentile; dots: individual points outside the whiskers) are shown. Sample size refers to the number of individual cochleae (**B**, **C**, **E**, and **G**), peaks (**D**), or waves (**F**) analyzed. Groups were compared with 1-way ANOVA with Dunnett’s test for multiple comparisons against control for each condition, with *P* values as indicated underneath each graph. Additionally, 2-tailed unpaired Student’s *t* test was performed to compare values at the 24-hour time point after noise exposure, as indicated in brackets with associated *P* values.

Though spontaneous ICS waves in supporting cells of the mature-hearing cochlea were scant, noise exposure elicited increased ICS activity (Supplemental Video 5 and Figure 6A). ICS waves propagated across and between all supporting cell types: phalangeal, inner and outer pillar, Deiters’, and Hensen’s cells. Exposure to 8–16 kHz octave-band noise at 98 and 106 dB SPL, which elicit TTS with cochlear synaptopathy and PTS with hair cell death, respectively, both induced a significant increase in the number of cell-level Ca^2+^ transients as well as organ-level ICS waves within 1 hour of initiation of noise (peaks/s [mean ± SEM]: 98 dB, 1.90 ± 0.16; 106 dB, 1.62 ± 0.39; unexposed, 0.75 ± 0.15; *P* = 0.0036, 1-way ANOVA; waves/s: 98 dB, 0.20 ± 0.03; 106 dB, 0.11 ± 0.02; unexposed, 0.07 ± 0.02; *P* = 0.0046, 1-way ANOVA; Figure 6, B and C). Twenty-four hours after completion of a full 2-hour noise exposure, cochleae exposed to 106 dB SPL noise, which ultimately causes hair cell death, had persistent Ca^2+^ peak activity (1.62 ± 0.39 peaks/s, 0.25 ± 0.05 waves/s), whereas those exposed to 98 dB SPL noise did not (1.28 ± 0.26 peaks/s, 0.12 ± 0.03 waves/s). For both noise exposure levels, the decay time of the Ca^2+^ transients in these supporting cells, which is a measure of persistent cytosolic Ca^2+^ elevation, was not significantly elevated (Figure 6, D and E). At the ICS wave level, cochleae exposed to 106 dB SPL noise had significantly longer distance of ICS wave propagation at both time points compared with control as well as 98 dB–exposed cochleae (Figure 6, F and G). Taken together, these findings demonstrate that noise exposure induces ICS wave activity in cochlear supporting cells and that this ICS-related cytosolic Ca^2+^ elevation is more persistent and extensive in cochleae exposed to the louder 106 dB SPL noise dose.

### Cochlear hair cells are not responsive to noise or ATP but demonstrate cytosolic Ca^2+^-associated cell death after noise exposure.

**Figure 7:**
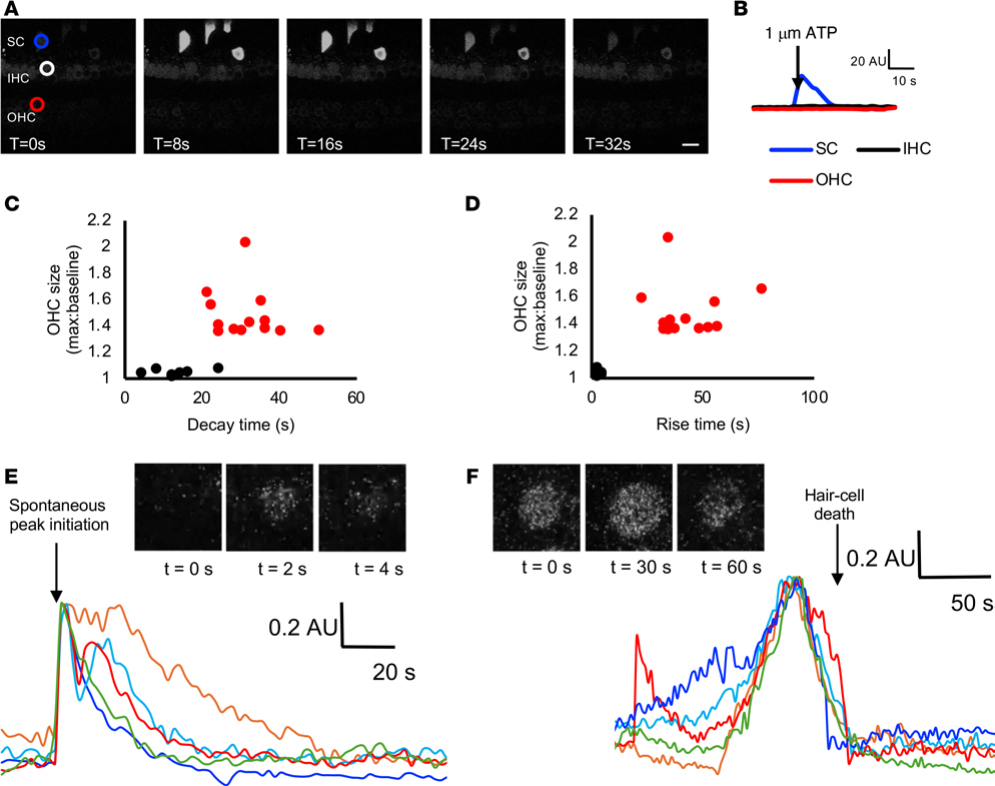
Live imaging of Myo15Cre-GCaMP6f adult cochlea. (**A** and **B**) In the adult cochlea, no spontaneous Ca^2+^ peak activity is seen in hair cells. Though an inner-sulcus supporting cell (SC, blue) with off-target expression of GCaMP6f responded to ATP (applied after the leftmost image) with a Ca^2+^ peak, neither an inner hair cell (IHC, white/black) nor an outer hair cell (OHC, red) was responsive. Time interval between successive images was 8 seconds. Scale bar: 20 μm. Representative video also shown in Supplemental Video 6. (**C**–**F**) Noise-induced Ca^2+^ transients. In fluorescence traces from OHCs from cochleae exposed to 106 dB noise, cytosolic Ca^2+^ transients were observed. Rise time (time from baseline to half-maximum, in seconds) (**D**) and decay time (**C**) for 20 Ca^2+^ transients was plotted against OHC maximum size relative to baseline, demonstrating 2 populations of transients: “slow” transients (red) that were associated with OHC swelling and fragmentation, and “fast” transients (black) that were not. Individual traces of fast (**E**) and slow (**F**) OHC transients are shown, with insets depicting still images of these events corresponding to video shown in Supplemental Video 6. Traces are normalized to maximal peak amplitude and aligned at the time of spontaneous peak initiation (**E**) or the time of hair cell fragmentation (**F**). AU, arbitrary units.

Adult cochlear hair cells displayed no spontaneous Ca^2+^ transients (Supplemental Video 3). Unlike neonatal hair cells, adult hair cells were not even responsive to external ATP. Application of 1 μM ATP elicited a robust Ca^2+^ transient in IS supporting cells with off-target expression of GCaMP6f, but not the adjacent hair cells (Figure 7, A and B). Exposure to 106 dB SPL noise elicited Ca^2+^ transients in OHCs (Supplemental Video 6). These transients could be clearly differentiated into 2 types based on rise and decay kinetics and cross-sectional area of the associated OHCs (Figure 7, C and D). Seven of 80 OHCs (8.8% of all OHCs, rate of 3.5 transients/min) exhibited “fast” transients with rapid onset and decay back to baseline, and they did not show changes in hair cell morphology (Figure 7E), though the decay times (27.2 ± 6.8 s [mean ± SEM]) were significantly longer than those seen for Ca^2+^ transients in supporting cells (2.52 ± 0.06 s for 106 dB noise exposure; Figure 6D, *P* < 0.0001 compared with hair cells). Thirteen of 80 OHCs (16.3% of all OHCs, rate of 6.5 transients/min) exhibited “slow” Ca^2+^ transients that occurred over an even longer time course and preceded hair cell swelling and fragmentation consistent with cell death (Figure 7F).

## Discussion

In this study, we used a mature-hearing, physiologically relevant model of NIHL to evaluate critical components of ER stress — activation of the UPR and alterations of Ca^2+^ homeostasis within hair cells and supporting cells — in the cochlear response to acoustic overstimulation. We found that the UPR is indeed activated immediately after multiple levels of noise exposure, peaking within 2 hours (Figure 2), with a shift toward the proapoptotic PERK/CHOP pathway only with the 106 dB noise-exposure level that causes PTS and hair cell death (Figure 1). Our findings corroborate and extend prior results in a physiological model of NIHL. Early upregulation of UPR genes has been suggested in cell-specific RNA-Seq analyses of noise-exposed mice (30), with specific genes — *Ddit3* and *Cebpb*, in particular — significantly upregulated after 105 dB SPL exposure. Some inconsistencies exist, however; for example, in our prior study, 106 dB noise exposure induced upregulation of *Chop*, *BiP*, and *S-Xbp1* in FVB mice, whereas in the current study, only *Chop* and *BiP* were increased in CBA/J mice, implying strain differences. The aforementioned RNA-Seq atlas (30) was obtained from Ai14 mice, and the Ca^2+^ imaging performed in the current study was performed in the C57BL/6 strain, which may have other strain-specific differences in UPR responses. Furthermore, the absence of upregulation of other proapoptotic factors, such as *Atf4*, in both our data and previous study (30) suggests complexity in noise-induced UPR regulation that must be understood. Our evaluation of UPR upregulation was limited to selected marker genes at the mRNA expression level; we did not explore more complex gene pathway alterations or downstream proteomic and phosphorylation changes that occur as part of the UPR and downstream apoptosis, which may be pursued in future studies.

Finally, we measured UPR gene expression in both male and female mice exposed to noise. Though we did not observe any sex-based difference in *Chop*/*S-Xbp1* ratio, there was a difference in CHOP expression after 106 dB SPL noise exposure between male and female mice, though this was not statistically significant after correcting for multiple comparisons (Figure 1). We have previously observed sex differences in UPR-targeted treatment of noise-induced cochlear synaptopathy (16), suggesting that there may be sex differences in the UPR response to noise that the current study is underpowered to detect.

After demonstrating that a genetically encoded Ca^2+^ indicator model that expresses GCaMP6f specifically in hair cells or supporting cells accurately reports cytosolic Ca^2+^ in neonatal cochleae (Figures 3 and 4), we studied the exact same noise-exposure models in mature-hearing, 7- to 8-week-old mice. Whereas neonatal, developing cochlea exhibited abundant spontaneous Ca^2+^ activity, especially ICS waves in supporting cells, both hair cells and supporting cells in the mature-hearing cochlea showed minimal spontaneous cytosolic Ca^2+^ transients or ICS waves, respectively (Figure 5). Spontaneous ICS activity in neonatal supporting cells has previously been shown to become quiescent at the onset of hearing (23), with suggestion of both spontaneous and evoked ICS waves in adult cochlea (16, 25); the current study extends these prior findings to show clearly that ICS activity can be potentiated by noise exposure. We found that, after noise exposure, supporting cells demonstrated increased ICS activity (Figure 6). Noise exposure of 106 dB, sufficient to cause PTSs and proapoptotic UPR activation, was associated with more prolonged and extensive ICS wave activity in the 24 hours after noise exposure. In contrast to prior findings of both “fast” and “slow” ICS waves in the adult cochlea (25), we only observed ICS activity comparable with the “fast” waves and similar to the ICS waves found in the neonatal cochlea. It is possible that the current recording configuration and duration was not optimized to detect these longer-duration and slower events.

In addition to the ICS activity in supporting cells, some hair cells demonstrated an increase in cytosolic Ca^2+^ preceding hair cell death (Figure 7). These transients were reminiscent of those observed in zebrafish hair cells exposed to aminoglycosides (13, 14), suggesting a similar role for cytosolic Ca^2+^ accumulation in the events immediately preceding hair cell death in the noise-exposed mammalian cochlea. Surviving hair cells generally did not demonstrate persistent elevation of cytosolic Ca^2+^ but instead showed only transient increases. Development of tools to simultaneously measure cytosolic, ER, and mitochondrial Ca^2+^ in adult cochlear hair cells is necessary to determine how these noise-evoked Ca^2+^ transients relate to prior findings in aminoglycoside-treated zebrafish neuromast hair cells.

These findings — that noise exposure immediately induces ICS waves in supporting cells, Ca^2+^ transients in hair cells, and UPR upregulation across the cochlea, with louder, PTS-associated noise specifically causing persistent ICS waves, UPR shift toward apoptosis, and cytosolic Ca^2+^ increases in hair cells, leading to their death — suggest that Ca^2+^ dysregulation and the UPR may constitute an early mechanism that can control subsequent hair cell death and PTS. The effectiveness of ISRIB, a small-molecule eIF2B activator that specifically reduces the proapoptotic arm of the UPR, in preventing NIHL (6), further supports the notion that the UPR is causally involved in NIHL and can be targeted for treatment. This work demonstrates the need to understand more precisely the timeline and interrelationship of these cellular events and additional molecular mediators that might serve as targets for treatment. In particular, it remains unknown exactly how ICS waves in supporting cells interact with Ca^2+^ transients in hair cells. Indeed, the relationship of ICS waves in astroglia and oligodendrocytes has been extensively studied in the central nervous system (17, 18, 33, 34), with myriad associations described between ICS activity and neuronal death, migration, and function but no universal “code” for how these ICS waves induce specific neuronal fates. The current adult cochlear live-imaging model may represent an opportunity to quantitatively assess the role of supporting cell ICS waves in influencing HC death. Do ICS waves in supporting cells induce hair cell Ca^2+^ transients and, subsequently, cause hair cell death? Or do dying hair cells induce ICS waves in the surrounding supporting cells? Indeed, the notion that ICS waves may be triggered by hair cell damage is supported by studies on ICS waves in neonatal cultures (19, 21), where mechanical trauma, laser ablation of hair cells, or neomycin treatment induced ICS waves and ERK1/2 activation in the supporting cells through which the ICS waves propagated. This ERK activation had further downstream effects on sensory epithelium remodeling and health of surrounding hair cells, illustrating the potential for ICS wave activity to modulate death and survival in the cochlea.

Alternatively, the opposite relationship is possible — ICS waves, which propagate in part through paracrine signaling mediated by ATP released by supporting cells (20), may trigger Ca^2+^ transients in adjacent hair cells. Whereas isolated transients may be tolerated by the hair cells, more intense or persistent ICS activity and ATP release may induce greater ER Ca^2+^ release in hair cells, thereby triggering hair cell death, either through ER Ca^2+^ depletion and the UPR or mitochondrial Ca^2+^ overload and oxidative stress. ATP is elevated in the endolymph after noise exposure (35), and targeting of purinergic receptor signaling has been proposed as a therapeutic strategy for NIHL (36). We found in this study that exogenous ATP induced robust cytosolic Ca^2+^ transients in neonatal hair cells but had no effect on hair cells in the mature-hearing cochlea. This may reflect changes in purinergic receptor expression or intracellular Ca^2+^ homeostasis over the course of hair cell development (37, 38); additionally, Ca^2+^ homeostasis is tightly regulated in hair cells between cytosolic buffering and highly regulated transfer between cytosol, ER, and mitochondria (12), and changes in cytosolic Ca^2+^ alone do not sufficiently predict cytotoxicity relating to downstream ER and mitochondrial effects (14). We observed that OHCs in noise-exposed cochleae did exhibit slow cytosolic Ca^2+^ accumulation that accompanies OHC swelling and fragmentation, consistent with OHC death, but we did not independently and concurrently ascertain cell death in this model. Findings in this study are limited the ex vivo preparation, in which critical physiologic features such as the endocochlear potential and ionic separation of the cochlear scalae are not maintained. Specific limitations of the current experimental setup — use of a water-immersion objective and bath application of drugs — may also cause optical artifacts that limit observation of fast and/or small responses. Development of an acoustically stimulated, active preparation of the mouse cochlea that provides an endocochlear potential and scalar separation, as has been described for the gerbil cochlea (39), would be valuable for further investigation of the precise interplay between ICS waves, ATP, subcompartmental Ca^2+^ homeostasis, and hair cell death in the mature, hearing cochlea.

The broad involvement of disorders of Ca^2+^ homeostasis, the UPR, and mitochondrial stress in genetic (12) and acquired (6, 7, 14) hearing loss highlight the need for further study to understand the underlying mechanisms in physiologically relevant disease models. In this study, we did not comprehensively evaluate all potential pathways by which noise exposure could induce changes in subcellular-compartment Ca^2+^ homeostasis and the associated downstream stress mechanisms. Specifically, we did not assess mitochondrial Ca^2+^ or stress pathways. However, our findings implicating cytosolic Ca^2+^ and the UPR in a consistent model of NIHL in mature-hearing mice does provide strong evidence for their involvement in the pathophysiology of NIHL. Understanding whether ICS waves are directly induced by noise and secondarily cause Ca^2+^ dysregulation in hair cells, or whether ICS waves are a response to hair cell injury that then subsequently helps to determine hair cell fate, is critical in order to identify targets for treatment.

In conclusion, we found that UPR activation and perturbations in cytosolic Ca^2+^ homeostasis in hair cells and supporting cells are involved in the cochlea’s early response to acoustic overstimulation. Given the critical role of the UPR, ICS waves, and cellular Ca^2+^ homeostasis in stress responses and subsequent cell fate, these findings suggest that targeting these pathways could be successful in treating NIHL. Further investigation into the specific mechanisms linking hair cell and supporting cell Ca^2+^ homeostasis and the UPR are necessary to more precisely identify targets for treatment.

## Methods

### Sex as a biological variable.

Our study examined male and female animals, and similar findings are reported for both sexes.

### Mouse models and cochlear preparation.

Sox2Cre (The Jackson Laboratory, 008454) or Myo15Cre (15) mice were bred with Ai95D mice (The Jackson Laboratory, 028865) for expression of GCaMP6f in supporting or hair cells, respectively. For WT noise exposures, 7- to 8-week-old CBA/CaJ mice (The Jackson Laboratory, 000654) were used. P3–P5 neonatal cochlear explant cultures were established as described (6). Briefly, P3–P5 mice were decapitated, temporal bones were extracted, and cochlear ducts were removed and plated on glass cover slips coated in Cell-Tak (Corning, 354240) with the apical surface of the epithelium facing up. Tectorial membrane was left intact, and cochleae were cultured overnight in DMEM supplemented with 10% FBS at 37°C in 5% CO_2_. Cultures that were grossly intact and remained fully adherent to the coverslip without sign of contamination were used for live imaging after overnight culture.

For live imaging in juvenile, mature-hearing cochleae, explant preparation was adapted from prior studies (25, 26, 31, 40); 7- to 8-week-old mice were euthanized with CO_2_ and decapitated immediately after noise exposure. The temporal bone was extracted from the skull by removal of the auditory bulla and then placed in ice-cold HBSS. Soft tissue and ossicles were removed with fine forceps, and the otic capsule was mounted on a custom 3D-printed slide fixated in a hole on a plastic coverslip. Fresh HBSS was applied, and the bone covering the apical turn of the cochlea was removed, preserving the membranous labyrinth and exposing the helicotrema. Reissner’s membrane was removed, and the preparation was used immediately for imaging. Time from euthanasia to imaging averaged less than 10 minutes. In all cases, only 1 cochlea was used per animal for live imaging.

Resonance (for Sox2Cre-GCaMP6f neonate) or line-scanning (for all other models) confocal imaging was performed on an upright Nikon A1R confocal microscope using a 60× water-immersion objective (NIR Apo, 1.0 NA), with temperature and CO_2_ control using a stagetop incubator (Okolab). Optical sections in the *x*–*y* plane were recorded at 1× averaging, 1.2 AU pinhole, 1.1 dwell time, displaying the entire IS, OS, and hair cell regions in the middle cochlear turn (neonates) and apical cochlear turn (8–10 kHz region, juveniles).

For neonatal cultures, baseline imaging was performed for 3 minutes, followed by vehicle (media), thapsigargin (Tocris), and/or sodium orthovanadate (Calbiochem) application at *t* = 3 minutes and, for Myo15Cre-GCaMP6f only, 1 µM ATP (MilliporeSigma) at *t* = 5 minutes. All drugs were bath applied to the final indicated concentrations and not washed out. A total of 20 minutes of imaging was performed, with an interval between each successive image of 1 second for Sox2Cre-GCaMP6f and 2 seconds for Myo15Cre-GCaMP6f. For imaging of juvenile, mature-hearing mice, a single 10-minute continuous imaging session was performed, with an interval of 2 seconds.

### UPR marker gene quantification.

UPR marker gene expression was quantified in cochleae from noise-exposed animals as described (6). Briefly, at the indicated times after completion of noise exposure, animals were euthanized and cochleae were harvested onto dry ice. Both cochleae from a single animal were pooled into a single specimen. Cochlear RNA was isolated from mice exposed to noise, or unexposed, using TRIzol Reagent (Thermo Fisher Scientific, 15596026). RNA quality was determined using a spectrophotometer and was reverse transcribed using Superscript IV VILO master mix (Thermo Fisher Scientific, 11756050). Expression levels of 3 UPR markers (*BiP*, indicative of UPR activation; *Chop*, correlated with proapoptotic activity of the UPR; and *S-Xbp1*, associated with prohomeostatic activity of the UPR) as well as *Gapdh* (as reference) were measured by qPCR and quantified against *Gapdh* and unexposed controls using the 2^–ΔCt^ method (Supplemental Figure 6). Samples with inadequate *Gapdh* levels (defined as Ct > 24) were excluded from analysis. The ratio of *Chop* over *S-Xbp1* was used as a marker of the proapoptotic state of the UPR (15).

In a separate experiment, mRNA was isolated identically from noise-exposed and unexposed mouse cochlea, and cDNA was tested using a real-time RT^2^ Profiler PCR Array (UPR Panel, QIAGEN, PAMM-089Z) in combination with RT^2^ SYBR Green qPCR Mastermix (QIAGEN, 330529). The PCR was run on a Bio-Rad CFX 384 real-time PCR cycler. Ct values were exported, and data analysis was conducted using the GeneGlobe Data Analysis Center (QIAGEN). Fold-change/regulation was calculated using the 2^–ΔCt^ method. Student’s 2-tailed *t* test was used to compare 2^–ΔCt^ values for each gene in control versus noise-exposure conditions.

### Image processing.

Ca^2+^ fluorescence measurements were performed on regions of interest (ROIs) slightly larger than a cell, as done previously (6). Briefly, images were reoriented such that the hair cells were parallel to the bottom of the image. In total, 325 ROIs, each 56 *×* 56 pixels (11 × 11 μm) in area, were defined (ImageJ; NIH), and mean fluorescence intensity was measured for every ROI at each time point. Ca^2+^ peak activity from each ROI-specific fluorescence time course was captured using a custom in-house script (Matlab, R2023a). Threshold for detection of a Ca^2+^ peak was set at 10 times the SD of the baseline fluorescence measurement.

### Noise exposure and auditory testing.

For NIHL induction, mice were exposed to 94, 98, or 106 dB SPL 8–16 kHz octave-band noise in a custom-built reverberant chamber as described (6), which respectively cause no hearing loss (94 dB), TTS with cochlear synaptopathy (98 dB) (16), and PTS with hair cell death (106 dB) (6). Hearing was tested in mice by measuring auditory brainstem response (ABR) thresholds in response to broadband tone pips at 8, 16, and 32 kHz in the sound field using a standard commercial system (RZ6, Tucker-Davis Technologies) in a soundproof chamber as described (6).

### Statistics.

Prior to analysis, data distribution was assessed for normality with the Shapiro-Wilks test. For normally distributed data, outliers were removed (ROUT method, *q* = 1%). For comparison between treatment groups, we used 2-tailed Student’s *t* test (for normally distributed data) or Mann-Whitney rank-sum test (for nonnormally distributed data) for comparisons between 2 groups, or 2-way ANOVA, followed by post hoc Dunnett’s multiple-comparison tests, for comparisons between > 2 groups. Unless otherwise mentioned, results are presented as means ± SEM with sample sizes and *P* values between designated comparison groups as indicated in the figure legends, with *P* < 0.05 as significant and lower *P* values indicated for specific comparisons. Statistical analyses were performed with GraphPad Prism 9.5.1. Sex was evaluated as a biological variable. UPR gene expression (Figure 1) and ABR thresholds (Supplemental Figure 1) after different noise-exposure levels were tested for male and female mice, and no significant differences found (unpaired 2-tailed *t* test with adjustment for multiple comparisons; Bonferroni correction for 3 simultaneous comparisons for 3 noise-exposure levels). Because no differences were seen in these core measures between sexes, all subsequent analyses used pooled male and female mice.

### Study approval.

This study was approved by the IACUC of the UCSF (AN1999783-00).

### Data availability.

All underlying data and supporting analytic code used in this study are either present in the published manuscript or the accompanying Supporting Data Values supplement or will be shared upon reasonable request made by email to the corresponding author.

## Author contributions

Initial design was contributed by YP, JL, EHS, and DKC. Experimental and ethical oversight and funding were contributed by EHS and DKC. Experimental contributions were contributed by YP, JL, NIM, IRM, PS, and DKC. Data and statistical analysis was contributed by YP, JL, NIM, IRM, PS, and DKC. Manuscript drafting was contributed by YP, JL, and DKC. Manuscript review and approval were contributed by all authors. YP and JL are co–first authors, and EHS and DKC are co–senior authors. This work was a collaboration between groups focused on cell biology and genetics (conducted by JL and EHS) and auditory physiology and imaging (conducted by YP, NIM, IRM, PS, and DKC). Because the physiology work constituted > 50% of the actual content in the study, YP is listed first among co–first authors and DKC is listed last among the co–senior authors.

## Supplementary Material

Supplemental data

Supplemental video 1

Supplemental video 2

Supplemental video 3

Supplemental video 4

Supplemental video 5

Supplemental video 6

Supporting data values

## Version 1 12/11/2024

In-Press Preview

## Version 2 01/23/2025

Electronic publication
