# Supplementary material for: Noise induces Ca2+ signaling waves and Chop/S-Xbp1 expression in the hearing cochlea
Source: JCI Insight. 2024 Dec 10;10(2):e181783. doi: 10.1172/jci.insight.181783 (PMC11790025; doi:10.1172/jci.insight.181783)
Supplement: Supplemental data [file jciinsight-10-181783-s146.pdf]

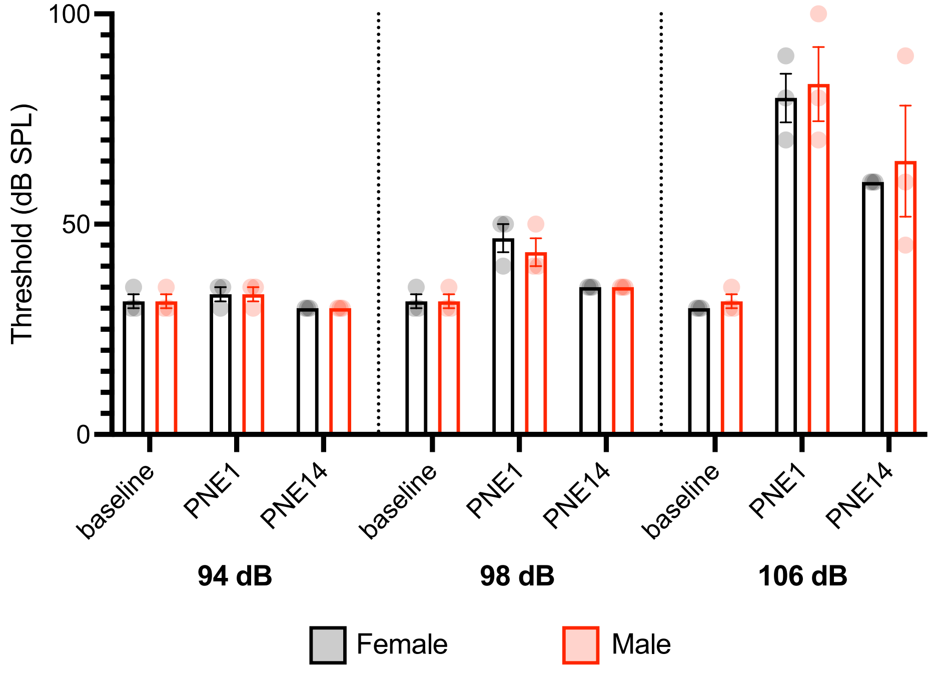

Supplemental Figure 1. Noise-induced hearing loss in CBA/J mice. 8-week-old wild-type female (black) and male (red) CBA/J mice were exposed to 8-16 kHz octave-band white noise at the indicated sound-pressure levels (dB SPL), and auditory brainstem response (ABR) thresholds recorded in response to click stimuli at the indicated days post-noise-exposure (PNE). Means ± sem for N=3 mice are shown, with individual data as dots.

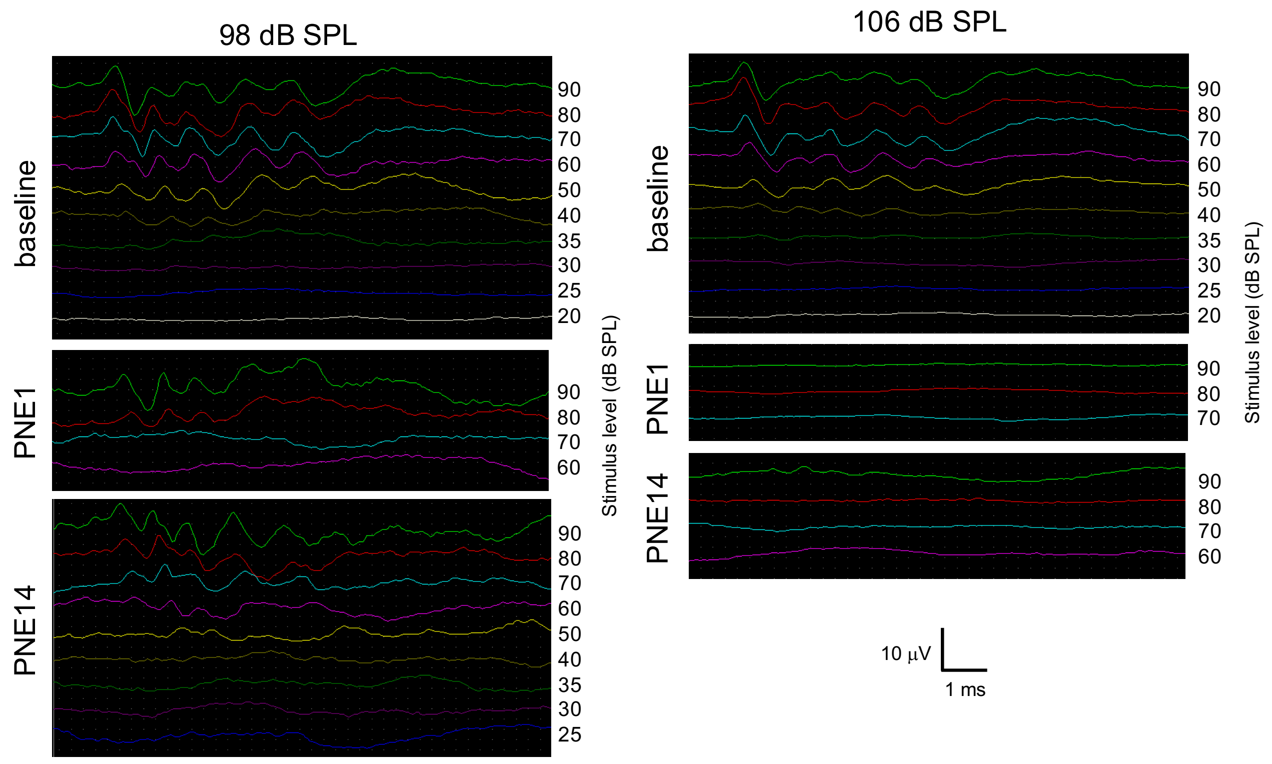

Supplemental Figure 2. Auditory brainstem response (ABR). Representative auditory brainstem response (ABR) traces) from mice exposed to 98 dB SPL (left) or 106 dB SPL (right) 8-16 kHz octave-band noise for 2h and tested at baseline (pre-exposure) and post-noise-exposure days 1 (PNE1) and 14 (PNE14).

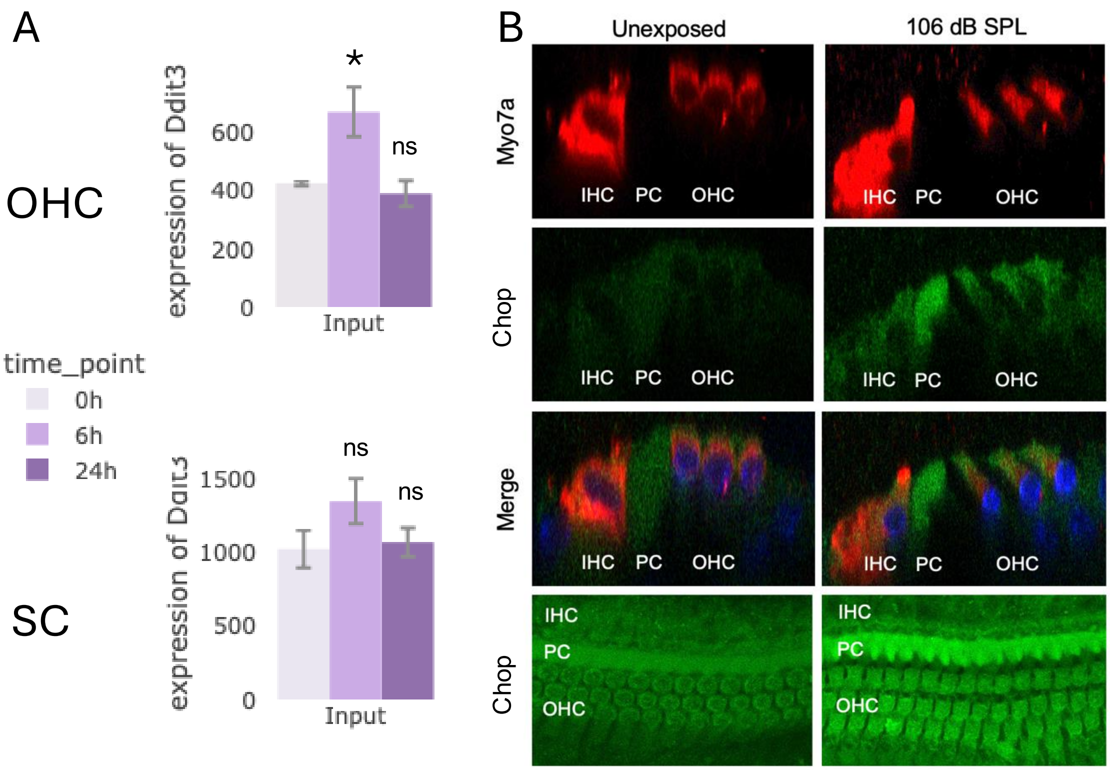

Supplemental Figure 3. *Chop*/*Ddit3* expression in cochlear cells. RNA levels for *Chop*/*Ddit3* in outer hair cells (OHC, top) and supporting cells (SC, bottom) from mice exposed to 105 dB SPL noise were extracted from existing RNASeq data from purified, Ribotagged OHCs and SCs (**A,** (ref. 30 and umgear.org). Means ± SD are presented. *, p<0.01; ns, not significant compared with 0h condition (two-tailed unpaired t test. **B.** Immunohistochemistry was performed against Chop (green) and Myo7a (red, a HC marker) and confocal microscopy performed on whole-mount cochleae from unexposed mice (left) and mice exposed to 106 dB SPL noise (right). Noise exposure increased Chop immunofluorescence in inner and outer HCs (IHC, OHC) and inner pillar cells (PC). Rows 1-3 represent sections across the organ of Corti reconstructed from confocal stacks, which are shown as maximum intensity projections in Row 4.

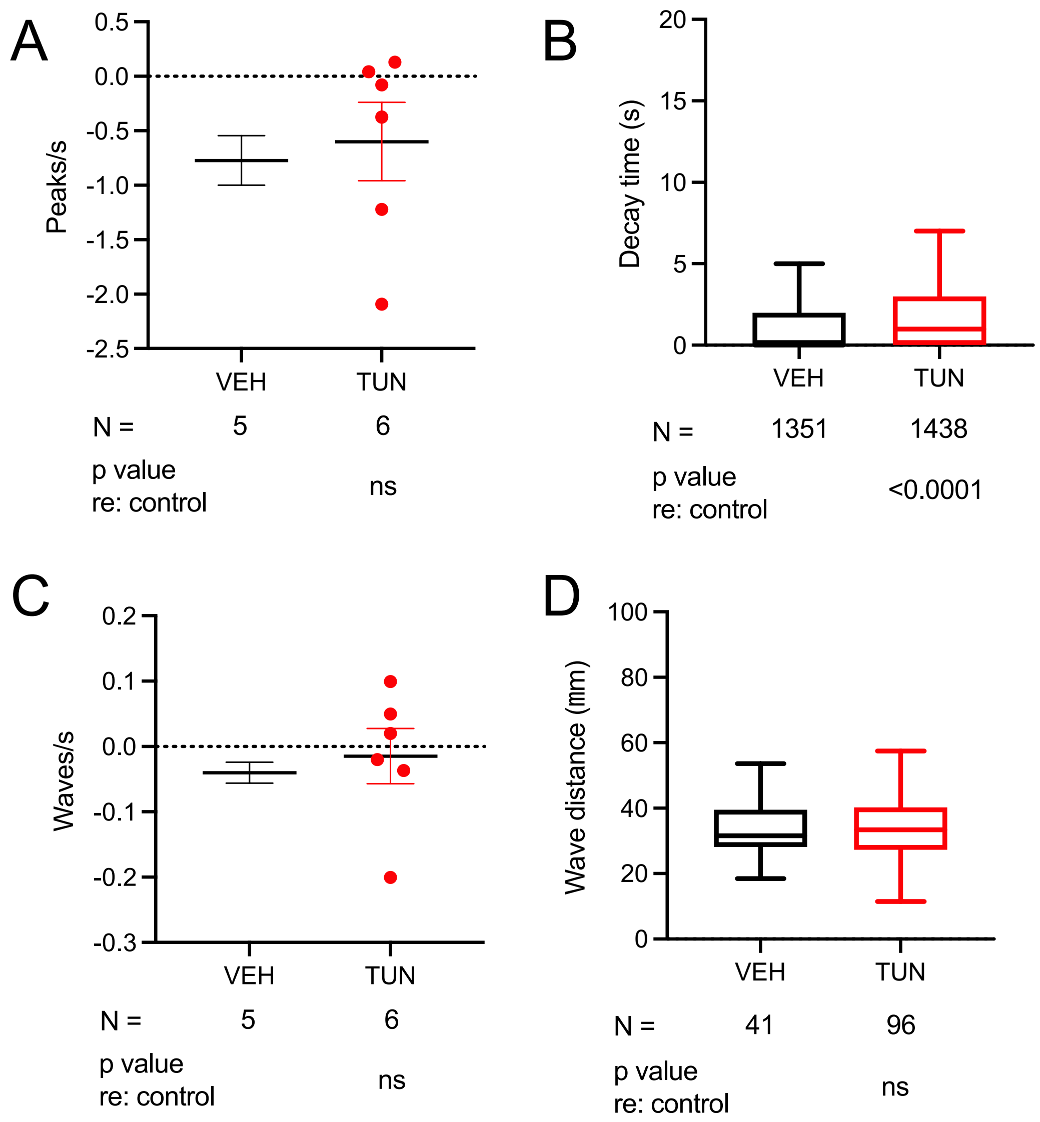

Supplemental Figure 4. Tunicamycin in ICS. A. Change in number of Ca^{2+} peaks per second after drug treatment. No difference in change in frequency of Ca^{2+} peaks from baseline to drug treatment is seen between vehicle (VEH, black) and tunicamycin (TUN, red). **B. Ca^{2+} decay time with drug treatment.** Peak decay time demonstrated significant prolongation of return to baseline Ca^{2+} levels in the presence of TUN. **C. Change in number of ICS waves per second after drug treatment.** No difference in change in frequency of ICS from baseline to drug treatment is seen between vehicle (VEH, black) and tunicamycin (TUN, red). **D. ICS wave distance propagation with drug treatment.** No difference in ICS wave propagation was seen

between VEH and TUN conditions. **A,C:** Means ± SEM, with individual cochlea-level values as dots. Groups were compared with one-way ANOVA. **B and D:** Tukey plots (box: 1st quartile/median/3rd quartile; whiskers: 10th and 90th percentile; dots: individual points outside the whiskers) representing all peaks **(B)** or waves **(D)** measured under the indicated conditions. P values are as indicated for pairwise comparisons versus control (VEH) on 2-tailed unpaired Student's t test. ns, not significant.

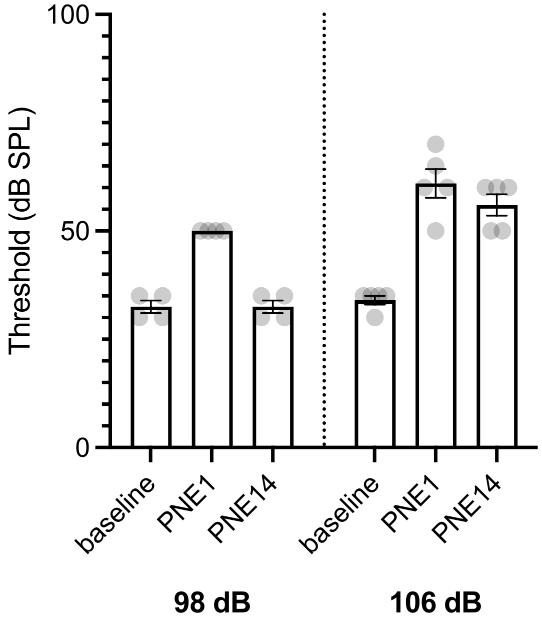

Supplemental Figure 5. Hearing in GcAMP mouse. Sox2Cre-GcAMP mice had normal hearing at baseline and exhibited appropriate temporary threshold shift (TTS) and permanent threshold shift (PTS) in response to 2h exposure to 8-16 kHz octave-band noise at 98 or 106 dB SPL, respectively. Means ± sem of auditory brainstem response (ABR) thresholds to click stimuli at the indicated days post noise exposure (PNE) are shown, with individual data in gray dots. N = 4 (98 dB) and 5 (106 dB).

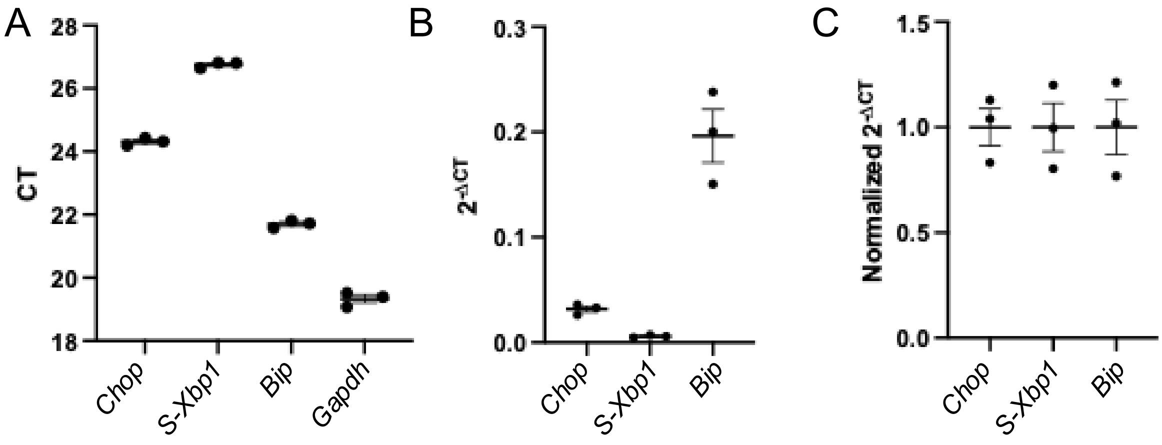

Supplemental Figure 6. qPCR measurement of UPR marker genes. **A.** qPCR was performed in mRNA isolated from whole cochleae of mice, in this case under control, non-noise exposed conditions. Cycle threshold (CT) was measured for 3 marker genes (*Chop*, *S-Xbp1*, and *BiP*) together with reference (*Gapdh*). **B.** Marker gene expression was then adjusted against *Gapdh* reference using the $2^{-\Delta CT}$ method. **C.** Finally, expression levels were normalized against control conditions to report fold-increase in expression re: control, as reported in Figures 1 and 2.

Supplemental Video 1. Supporting-cell Ca^{2+} activity in the neonatal cochlea. Spontaneous Ca^{2+} activity was visualized by resonance confocal microscopy in supporting cells of a cochlear culture from a neonatal Sox2Cre-GcAMP mouse. Each successive frame represents a 1-s time interval; this video shows 195 s from a 600-s recording, representative of n = 7 animals. IS, inner sulcus; HC, hair-cell region; OS, outer sulcus. Scale bar: 20 μm.

Supplemental Video 2. Hair-cell Ca^{2+} activity in the neonatal cochlea. Ca^{2+} activity was visualized by line-scanning confocal microscopy in hair cells of a cochlear culture from a neonatal Myo15Cre-GcAMP mouse. At t = 88 s, 1 μm ATP was added to the bath. Each successive frame represents a 2-s time interval; this video shows 184 s from a 600-s recording, representative of n = 3 animals. IS, inner sulcus; IHC, inner hair cells; OHC, outer hair cells; OS, outer sulcus. Scale bar: 20 μm.

Supplemental Video 3. Hair-cell Ca^{2+} activity in the mature, hearing cochlea. Ca^{2+} activity was visualized by line-scanning confocal microscopy in hair cells of a cochlear explant from an 8-week-old Myo15Cre-GcAMP mouse. At t = 120 s, 1 μm ATP was added to the bath. Each successive frame represents a 2-s time interval; this video shows 246 s from a 600-s recording, representative of n = 3 cochleae. IS, inner sulcus; IHC, inner hair cells; OHC, outer hair cells; OS, outer sulcus. Scale bar: 20 μm.

Supplemental Video 4. Supporting-cell Ca^{2+} activity in the mature, hearing cochlea. Spontaneous Ca^{2+} activity was visualized by line-scanning confocal microscopy in supporting cells of a cochlear explant from an 8-week-old Sox2Cre-GcAMP mouse. Each successive frame represents a 2-s time interval; this video shows 196 s from a 600-s recording,

representative of n = 15 cochleae. IS, inner sulcus; HC, hair-cell region; OS, outer sulcus. Scale bar: 20 μm.

Supplemental Video 5. Supporting-cell Ca^{2+} activity in the noise-exposed mature, hearing cochlea. Spontaneous Ca^{2+} activity was visualized by line-scanning confocal microscopy in supporting cells of a cochlear explant from an 8-week-old Sox2Cre-GcAMP mouse immediately after exposure to 98 dB SPL, 8-16-kHz noise. Each successive frame represents a 2-s time interval; this video shows 196 s from a 600-s recording, representative of n = 4 cochleae. IS, inner sulcus; PC, pillar cells; DC, Deiter's cells; OS, outer sulcus. Scale bar: 20 μm.

Supplemental Video 6. Hair-cell Ca^{2+} activity in the noise-exposed mature, hearing cochlea. Spontaneous Ca^{2+} activity was visualized by line-scanning confocal microscopy in supporting cells of a cochlear explant from an 8-week-old Myo15Cre-GcAMP mouse immediately after exposure to 106 dB SPL, 8-16-kHz noise. At t = 106 s, 1 μm ATP was added to the bath. Each successive frame represents a 2-s time interval; this video shows 200 s from a 600-s recording, representative of n = 4 cochleae. Red circle indicates an outer hair cell (OHC) demonstrating an example of a "slow" Ca^{2+} transient accompanied by cell swelling, fragmentation, and death; yellow circle indicates an OHC demonstrating an example of a "fast" Ca^{2+} transient that is not accompanied by cell death. IS, inner sulcus; IHC, inner hair cells. Scale bar: 20 μm.
